# Can Brief Cognitive or Medication Management Tasks Identify the Potential for Dependence in Instrumental Activities of Daily Living?

**DOI:** 10.3389/fnagi.2020.00033

**Published:** 2020-02-20

**Authors:** Timothy S. Marks, Gordon M. Giles, Muhammad O. Al-Heizan, Dorothy F. Edwards

**Affiliations:** ^1^Department of Kinesiology-Occupational Therapy, The University of Wisconsin–Madison, Madison, WI, United States; ^2^Department of Occupational Therapy, Samuel Merritt University, Oakland, CA, United States; ^3^Department of Rehabilitation Sciences, King Saud University, Riyadh, Saudi Arabia

**Keywords:** functional cognition, instrumental activities of daily living, screening, Medi-Cog-R, performance assessment

## Abstract

**Background and Objectives:** The identification of functional performance deficits is critical to the community independence of older adults. We examined whether a combined cognitive and performance-based medication management measure would be able to better classify an individual’s functional cognitive status and potential for instrumental activities of daily living (IADL) impairment than either measure alone.

**Research Design and Methods:** Community-dwelling adults age 55 and older (*n* = 185) were administered the Mini-Cog, the Medication Transfer Screen-Revised (MTS-R), a combination measure the Medi-Cog-Revised (Medi-Cog-R), the Performance Assessment of Self-Care Skills (PASS) Checkbook Balancing and Shopping tasks (PCST), additional cognitive screening measures, and a self-report daily living scale. Receiver operating characteristic (ROC) curve analyses were computed for the Mini-Cog, MTS-R and the Medi-Cog-R using the PCST performance as the criterion measure. The area under the curve (AUC), sensitivity, and specificity were computed for each measure.

**Results:** The Medi-Cog-R most accurately identified individuals as impaired on the PCST. An AUC statistic of 0.82 for the Medi-Cog-R was greater than either the Mini-Cog (0.75) or the MTS-R (0.73). The Medi-Cog-R demonstrated a sensitivity of 0.71 and a specificity of 0.78 in classifying individuals with impaired IADL as measured by the PCST.

**Discussion and Implications:** The Mini-Cog, the MTS-R, and the Medi-Cog-R all show discriminant validity, but the combined measure demonstrates greater sensitivity and specificity than either component measure alone in identifying IADL impairment. The Medi-Cog-R appears to be a useful screening measure for functional cognition and can be used to prompt further assessment and intervention to promote community independence.

## Introduction

Functional cognition has been defined as the ability to use and integrate thinking and performance skills to accomplish complex everyday activities, including instrumental activities of daily living (IADL; Giles et al., 2017). Some IADL are considered essential for community living such as health management and maintenance (e.g., nutrition, medication management), financial management, and community mobility skills (e.g., driving, using public transit; American Occupational Therapy Association, 2014). Historically, clinicians have observed clients’ attempts to perform complex functional tasks in order to infer general IADL competency (Skidmore, 2017). However, in the simplified environments of the acute or post-acute care (PAC) settings, IADL performance is not typically directly observable (Giles et al., 2017), and other methods of determining the presence of deficits significant enough to impair IADL are needed (Schmitter-Edgecombe et al., 2011; Gold, 2012). Attempts to predict IADL deficits from neuropsychological measures have had limited success (Marcotte et al., 2010; Schmitter-Edgecombe et al., 2011; Gold, 2012), and informant reports often underestimate the need for assistance (Jonas et al., 2011; Schmitter-Edgecombe et al., 2011). Self-report measures have also been shown to overestimate independence on IADL especially in individuals with new-onset conditions who may lack relevant experience of functional deficits or self-awareness (Nielsen et al., 2016). Therefore, clinicians may have inadequate knowledge of client post-discharge needs and clients may not be provided with adequate support during care transitions (Leland et al., 2019).

Impaired functional cognition is associated with IADL impairment (Moore et al., 2007; Puente et al., 2014; Wesson et al., 2016). In attempting to obtain ecologically valid data about a client’s ability to meet IADL demands in settings where these demands cannot be observed directly occupational therapists and others have developed performance-based measures of functional cognition (Giles et al., 2017; Skidmore, 2017). Such measures employ potentially familiar IADL tasks, the enactment of which can be influenced by client self-awareness, strategy use, and previously acquired performance skills. Performance-based tests of functional cognition have repeatedly been found to better predict real-world functioning and community independence than other forms of assessment (Puente et al., 2014; Wesson et al., 2016). Against this background, potential changes to the standard PAC assessment batteries being studied by the Centers for Medicaid and Medicare Services (CMS) in the USA in response to The IMPACT Act (Improving Medicare Post-Acute Care Transformation Act, 2014) have provided an opportunity to reconsider the screening tools used in PAC settings, but these considerations are also relevant for acute and community treatment settings (DeJong, 2017). CMS has identified functional cognition screening as an important domain for measurement consideration (Skidmore, 2017; Medicare Program, 2019) and has suggested that a task involving medication management might be optimal (RAND Corporation, 2017) as independent medication management is associated with community independence and is likely to have face validity for consumers (Royall et al., 2007). An effective screening tool to identify individuals at risk for hospital readmission would support hospitals and PAC settings in directing multifaceted interventions and resources to those who are at risk (Kripalani et al., 2014). The available performance-based tests of functional cognition were developed primarily for outpatient settings and often require 20 min or more to complete. This testing time makes them inappropriate for use in settings where testing time is limited (Baum et al., 2003; Fisher and Bray Jones, 2010; Morrison et al., 2015; Rogers et al., 2016). Rapidly administered screening measures are needed to identify individuals in need of further functional cognitive assessment (Edwards et al., 2019).

We hypothesized that a cognitive measure combined with a performance-based medication management measure would be able to better classify an individual’s functional cognitive status and potential for IADL impairment than either of the measures used alone. We examined the utility of two brief screening measures, the Mini-Cog (Borson et al., 2003), and a performance-based revision of the Medication Transfer Screen (MTS; Anderson et al., 2008), the Medication Transfer Screen-Revised (MTS-R) separately, and then examined their combination—the Medi-Cog-Revised (Medi-Cog-R)—to determine the most effective screening tool to identify individuals at risk for functional cognition impairment. The Mini-Cog is a widely used screening measure sensitive to cognitive impairments associated with a variety of diagnoses including dementia, diabetes, and heart failure, that has demonstrated clinical utility in primary care, acute care, and community settings (Sinclair et al., 2013; Kallumpuram et al., 2015; Patel et al., 2015). The Mini-Cog has also been used in combination with a medication transfer screening test to identify community-dwelling individuals at risk for medication mismanagement (Anderson et al., 2014). The MTS is a paper and pencil task that simulates the sorting of actual medications. It includes a week-long paper grid representing an actual pillbox with four sections per day to represent times when medications could be taken. Directions for four different medications are written above the grid and the participant is asked to insert numbers onto the grid in pencil that represent the number of medications to be taken at each designated time and day. The final requirement in the MTS requires the individual to report the total number of tallies for the entire day of Saturday. In this study, we made minor alterations to increase the performance demands of the MTS converting it from a paper and pencil to a practical task (MTS-R) by using pill bottles, fake medications and a 4 × 7 section medication organizer into which the fake medications are to be distributed. Due to this performance-based modification, the combined Mini-Cog and MTS-R named the Medi-Cog-R, differ from the original Medi-Cog which utilized the paper and pencil MTS. The original validation studies of the MTS focused only on determining participant’s medication management competency (Anderson et al., 2014), but we evaluated the Mini-Cog, MTS-R and the Medi-Cog-R for their ability to more broadly screen for impaired functional cognition with general implications for IADL performance. Currently, there are no gold standard screening assessments for functional cognition or IADL impairment (i.e., no one test can fully capture these complex and broad constructs). Rapid and easy to administer functional cognitive screening tests have the potential to increase the early detection of impairments that may interfere with IADL task performance after discharge. It should be noted that screening tests are not diagnostic and impairment on these screening tests does not necessarily mean that an individual has impaired functional cognition or that they will be unable to independently perform IADL tasks. However, if an individual demonstrates difficulties when completing a functional cognitive screening test, that information suggests that clinicians should further assess functional cognitive abilities in relation to the specific roles and required competencies for that individual’s life context.

A community sample was used for this pilot study, as individuals living in the community are the most likely to need to manage their own IADL (Gaugler et al., 2007). The combined number of cues on two subtests of the Performance Assessment of Self-Care Skills (PASS) Checkbook Balancing and Shopping tasks (PCST) was used as the criterion measure for these analyses because this score distinguished between older adults with mild cognitive impairment (MCI) and cognitively unimpaired older adults and is more complex than a simple screen (Rodakowski et al., 2014). The complete PASS battery contains 26 tasks, 14 of which include IADL with greater cognitive than physical demands. In a study by Rodakowski et al. (2014), eight of these cognitively challenging IADL (shopping, bill paying, checkbook balancing, bill mailing, telephone use, medication management, critical information retrieval, and small device repair) were analyzed to determine which tasks were the most psychometrically sound and which could differentiate between individuals with unimpaired cognitive function and those with MCI in a sample of 157 community-residing older adults. All eight tasks combined demonstrated a sensitivity of 0.75 and a specificity of 0.73 to differentiate individuals with unimpaired cognitive function vs. MCI. However, the PCST was found to be the most sensitive in classifying individuals with MCI, and together had the similar discriminative ability to all eight tasks combined. According to Rodakowski et al. (2014) the shopping and checkbook balancing tasks used in combination had a sensitivity of 0.70 and a specificity of 0.70 to differentiate individuals with normal cognitive function vs. MCI, suggesting that these two tasks could be used in lieu of the full battery without significantly sacrificing sensitivity or specificity. Additional support for the use of the PASS comes from a secondary analysis by Brown and Finlayson (2013) who compared the predictive validity of two self-report assessments of IADL and five existing PASS tasks (mobility in the house, medication routine, paying bills, shopping, telephone use) combined with two additional items (laundry and first aid) constructed using the PASS criteria for item development. The seven combined items contributed to the prediction of home care utilization while the self-report measures did not. When looking at the PASS task items individually, mobility in the house, paying bills, shopping, and laundry were the items that had statistically significant relationships with home care utilization. Thus, the Brown and Finlayson study provides additional support for the use of the PASS in predicting health services utilization in community-dwelling adults. These findings are consistent with recent analyses of Medicare Claims data linking IADL impairment with increased health care utilization (Strotmeyer and Ensrud, 2018).

The current study also aimed to determine the discriminant validity of three screening tests in a community sample with the goal of determining whether any of the measures had the potential to screen for functional cognitive impairments contributing to IADL difficulties. We examined the discriminant validity of these screening tests by comparing the scores of individuals classified as unimpaired or impaired to established cognitive screening measures and a self-report measure of ADL/IADL independence.

## Design and Methods

### Research Design

This cross-sectional observational study was approved by the Institutional Review Board of the University of Wisconsin–Madison. All participants provided written informed consent.

### Participants and Recruitment

A convenience sample of community-dwelling adults was recruited in Madison, Wisconsin and its environs (*n* = 185) using flyers posted in community settings. Additionally, in-person recruitment included researchers attending community events and word of mouth referrals from study participants. Inclusion criteria were: age 55 years or older, living independently in the community, and willingness and ability to read and write in English. Exclusion criteria were: age under 55 years, living in a skilled nursing or assisted living facility, and unable to hear, read, or write in English.

## Measures

### Mini-Cog

The Mini-Cog is a widely used two-part rapid screening measure that incorporates three-word recall and a clock-drawing test and takes 2 to 3 min to administer (Lam et al., 2011). When compared with the Mini-Mental State Exam (MMSE; Folstein et al., 1975) in a community-dwelling multiethnic sample the overall accuracy of classification for cognitive impairment was 83% for the Mini-Cog and 81% for the MMSE (Borson et al., 2005). Poor performance on the Mini-Cog was shown to be a marker of post-hospitalization risk and readmission (Patel et al., 2015). A cutoff score of 3 has been validated for dementia screening and a cutoff score of 4 is recommended for greater sensitivity (Borson et al., 2003; McCarten et al., 2011).

### Performance-Based Measures

#### Medication Transfer Screen-Revised

The MTS-R is a performance-based adaptation of the MTS (Anderson et al., 2008, 2014). In the MTS, individuals must follow written instructions and write numbers on a week-long grid to represent medications and answer a question about pill count. The MTS-R is a practical revision of the MTS in which the week-long paper grid has been replaced by a 4 × 7 section medication organizer and the four written medication instructions have been replaced by four pill bottles filled with fake pills and that have medication instructions written on the bottle. Instead of writing numbers onto the paper grid, individuals are given the pill bottles and must follow the instructions on the pill bottle labels to correctly distribute the pills into the medication organizer. For the last item of the test, instead of tallying the number of medications written onto the paper for Saturday as is required in the MTS, the individual must now look at the medication organizer for Saturday and count the number of fake medications distributed for that day and report this to the examiner. Scores for the MTS-R range from 0 to 5. In stepwise regression conducted with the MTS, Anderson et al. (2008) reported that the MTS significantly predicted the ability to fill medication after controlling for age (*p* < 0.001).

#### Medi-Cog-R

The Medi-Cog is a combination of the Mini-Cog and the MTS (Anderson et al., 2008, 2014; Lam et al., 2011). For this study we combined the five items Mini-Cog with the five items MTS-R, resulting in the 10 items Medi-Cog-R (Anderson et al., 2008).

#### Performance Assessment of Self-Care Skills

The PASS (Rogers et al., 2016) includes 26 items that measure ADL and IADL skills intended to assist clinicians in planning interventions, 14 of which are described as IADL items with a cognitive emphasis. The PASS Checkbook Balancing and Shopping tasks (PCST) have been found to be as sensitive as all of the PASS IADL tasks in discriminating between individuals with MCI and healthy older adults and were used for the current study (Rodakowski et al., 2014). PCST scores were based on the combined number of cues required for independence and adequacy on each task, with lower numbers indicating higher performance (fewer cues needed). Rodakowski et al. (2014) reported that this PCST scoring system had an area under the curve (AUC) of 0.80 (*p* < 0.001) and sensitivity of 0.70 and specificity of 0.70 in predicting cognitive status (unimpaired cognitive function or MCI), with a cut-point of eight cues.

### Neuropsychological Measures

#### Brief Interview of Mental Status

The Brief Interview of Mental Status (BIMS; Chodosh et al., 2008; Saliba et al., 2012) is a 15-point assessment that evaluates memory, orientation, and includes free and cued recall items. Possible scores range from 0 (no items correct) to 15 (all items correct). Scores of 12 or less are indicative of cognitive impairment. CMS has selected the BIMS for inclusion as a standard assessment in all PAC settings (Medicare Program, 2019).

#### Montreal Cognitive Assessment

The Montreal Cognitive Assessment (MoCA; Nasreddine et al., 2005) is a 30-point cognitive impairment screening tool found to have good internal consistency with a Cronbach’s alpha of 0.83. Nasreddine et al. (2005) administered the MoCA to three groups: persons with Alzheimer’s disease (AD), persons with MCI, and older adults without cognitive impairment. Sensitivity was high for identifying both AD and MCI in older adults (100% and 90% respectively) with specificity of 87%. Scores of less than 26 are indicative of cognitive impairment.

### Self-report ADL/IADL Measure

#### Alzheimer’s Disease Cooperative Study—Activities of Daily Living Inventory Scale

The AD Cooperative Study—Activities of Daily Living Inventory Scale (ADCS) consists of 23 self or informant report items designed to evaluate ADL and IADL performance in individuals enrolled in AD clinical trials (Galasko et al., 1997). The ADCS has good test-retest reliability, correlates with cognitive screening measures in individuals with MCI and AD and is sensitive to disease progression (Galasko et al., 1997; Doraiswamy et al., 2014; Cintra et al., 2017). Self-report and proxy ratings have been found to be highly correlated with the ADCS (Howland et al., 2017) which was used in this study as a self-report measure. Higher scores indicate greater independence.

#### Procedure

Occupational therapy graduate students from the University of Wisconsin–Madison performed all testing. Testing was conducted in quiet settings (i.e., private spaces in senior centers or senior co-housing), with the participant seated at a table. Participants completed a questionnaire documenting demographic information and brief health history. The test battery was administered in the following arbitrary order: Mini-Cog, MTS-R, BIMS, MoCA, PASS Checkbook Balancing and Shopping tasks, and the ADCS.

#### Statistical Analyses

Descriptive analyses for continuous data and frequency distributions for non-continuous demographic data were computed. Receiver operating characteristic (ROC) curve analyses were computed to determine the AUC for the two screening measures and their combination using the PCST as the criterion variable. The AUC statistics produced by the ROC analyses were used as an index of predictive validity in combination with the sensitivity and specificity coefficients for the Mini-Cog, MTS-R, and the Medi-Cog-R. Published criteria for the Mini-Cog and cutoff scores based on ROC analyses for the MTS-R and Medi-Cog-R were then used to dichotomize individuals into unimpaired and impaired groups for each screening measure. Independent groups *t*-tests were then used to compare the mean scores on the BIMS, MoCA, and the ADCS between unimpaired and impaired groups of the two screening measures and their combination. IBM SPSS Version 25 (IBM Corp, 2017) was used for all statistical analyses.

## Results

The assessment battery was administered to 185 community-dwelling participants age 55 and older. The Mean age of the participant was 70.68 (*SD* = 8.30) years old. The sample was relatively healthy, with an average of 1.21 reported chronic health conditions. The sample was predominantly female (76.2%) and white (80.4%). Table 1 presents the demographic characteristics of the sample and scores for each study measure.

**Table 1:** Demographic characteristics and scores on study measures.

Variable	Mean (SD)	Range
Age (years)	70.68 (8.30)	55–93
Chronic health conditions	1.21 (1.30)	0–7
Education (years)	15.17 (3.01)	8–27
BIMS	14.56 (0.90)	11–15
MoCA	23.90 (3.70)	14–30
Mini-Cog	3.99 (1.24)	0–5
MTS-R	4.02 (1.09)	1–5
Medi-Cog-R	8.01 (1.84)	3–10
PCST # of cues	10.34 (9.73)	0–48
ADCS	74.88 (4.68)	42–78
	N (%)	
Female	141 (76.2)	
White	148 (80.4)	

*Note. ADCS, Alzheimer’s Disease Cooperative Study, possible range 0–78; BIMS, Brief Interview of Mental Status, possible range 0–15; Medi-Cog-R, possible range 0–10; Mini-Cog, possible range 0–5; MoCA, Montreal Cognitive Assessment, possible range 0–30; MTS-R, Medication Transfer Screen-Revised, possible range 0–5; PCST, Performance Assessment of Self-care Skills Checkbook Balancing and Shopping Task, SD, Standard deviation*.

In order to examine the ability of the screening measures to identify individuals with IADL impairment, we computed a ROC analysis to determine the AUC and the sensitivity and specificity of the two screening measures and their combination using the PCST as the dependent measure. The Mini-Cog and MTS-R demonstrated AUC statistics of 0.75 and 0.73, respectively. The combined Medi-Cog-R resulted in a higher AUC value (0.82) than either of its constituent measures. We examined the sensitivity and specificity of the Mini-Cog using a cutoff score of 4, and optimal cutoff scores for the MTS-R and Medi-Cog-R were found to be 5 (MTS-R) and 9 (Medi-Cog-R), with scoring below the cutoff as indicative of impairment. The results of these analyses are presented in Table 2. A sensitivity of 0.71 and a specificity of 0.78 was obtained with a cut-off score of 9 on the Medi-Cog-R.

**Table 2:** ROC summary.

Test (cutoff)	AUC	Sensitivity	Specificity
Mini-Cog (4)	0.75	0.41	0.91
MTS-R (4)	0.73	0.33	0.91
MTS-R (5)	0.73	0.60	0.73
Medi-Cog-R (8)	0.82	0.51	0.90
Medi-Cog-R (9)	0.82	0.71	0.78

*Note. Scores below the cutoff point indicate impairment. Medi-Cog-R, possible range 0–10; Mini-Cog, possible range 0–5; MTS-R, Medication Transfer Screen-Revised, possible range 0–5*.

The cutoff scores were then used to classify participants into unimpaired or impaired groups for each of the Mini-Cog, MTS-R, and the Medi-Cog-R. Between groups, *t*-tests were computed to compare the scores of the unimpaired and impaired groups on the cognitive screening measures and the self-reported ADCS ADL/IADL scale. The result of these analyses for the Mini-Cog, the MTS-R, and the Medi-Cog-R are presented in Table 3. Individuals impaired on the Mini-Cog were significantly older than those classified as unimpaired (*p* = 0.045), however no significant age differences were found between the MTS-R and the Medi-Cog-R impaired and unimpaired groups. The number of chronic health conditions for individuals impaired on the Medi-Cog-R was slightly higher (*M* = 1.46, *SD* = 1.45) than those who were unimpaired (*M* = 1.07, *SD* = 1.19), this difference approached but did not reach significance (*p* = 0.060). Years of education differed significantly between impaired and unimpaired groups for each of the three screening measures. The means of years of education were significantly higher in the unimpaired group for each measure (see Table 3).

**Table 3:** Student’s independent Groups *t*-Tests comparing individuals unimpaired and impaired on the Mini-Cog (Cutoff = 4), MTS-R (Cutoff = 5), and Medi-Cog-R (Cutoff = 9).

	Mini-Cog unimpaired (*n* = 128)	Mini-Cog impaired (*n* = 57)	
	mean (SD)	mean (SD)	*t*_(1,183)_; *p* =
Age (years)	69.86 (7.83)	72.51 (9.08)	−2.02; *p* = 0.045
Chronic health conditions	1.09 (1.20)	1.49 (1.47)	−1.83; *p* = 0.070
Education (years)	15.66 (3.22)	14.05 (2.49)	3.34; *p* = 0.001
BIMS	14.69 (0.75)	14.26 (1.11)	2.63; *p* = 0.010
MoCA	25.31 (2.98)	20.72 (3.17)	9.50; *p*< 0.001
MTS-R	4.19 (1.06)	3.65 (1.06)	3.18; *p* = 0.002
Medi-Cog-R	8.91 (1.18)	6.00 (1.41)	13.56; *p*< 0.001
PCST # of cues	7.61 (7.46)	16.62 (11.40)	−5.38; *p*< 0.001
ADCS	75.75 (3.06)	72.91 (6.71)	3.06; *p* = 0.003
	MTS-R unimpaired (*n* = 79)	MTS-R impaired (*n* = 106)	
	mean (SD)	mean (SD)	*t*_(1,183)_; *p* =
Age (years)	70.65 (7.78)	70.75 (9.73)	−.07; *p* = 0.949
Chronic health conditions	1.08 (1.27)	1.58 (1.35)	−2.34; *p* = 0.021
Education (years)	15.67 (2.86)	13.71 (3.32)	3.87; *p*< 0.001
BIMS	14.61 (0.85)	14.40 (1.01)	1.45; *p* = 0.149
MoCA	24.62 (3.45)	21.83 (3.66)	4.75; *p*< 0.001
Mini-Cog	4.10 (1.17)	3.67 (1.37)	1.96; *p* = 0.054
Medi-Cog-R	8.68 (1.41)	6.10 (1.56)	10.57; *p*< 0.001
PCST # of cues	8.08 (7.77)	16.81 (11.78)	−4.73; *p*< 0.001
ADCS	75.18 (4.76)	74.00 (4.38)	1.51; *p* = 0.132
	Medi-Cog-R unimpaired (*n* = 91)	Medi-Cog-R impaired (*n* = 94)	
	mean (SD)	mean (SD)	*t*_(1,183)_; *p* =
Age (years)	69.99 (7.59)	71.88 (9.37)	−1.41; *p* = 0.161
Chronic health conditions	1.07 (1.19)	1.46 (1.45)	−1.90; *p* = 0.060
Education (years)	15.87 (2.95)	13.92 (2.98)	4.29; *p*< 0.001
BIMS	14.66 (0.86)	14.37 (0.94)	2.12; *p* = 0.035
MoCA	25.46 (2.88)	21.15 (3.38)	9.17; *p*< 0.001
Mini-Cog	4.64 (0.61)	2.84 (1.23)	11.31; *p*< 0.001
MTS-R	4.55 (0.66)	3.09 (1.07)	10.14; *p*< 0.001
PCST # of cues	6.40 (4.38)	17.59 (11.84)	−7.20; *p*< 0.001
ADCS	75.67 (4.049)	73.48 (5.37)	2.91; *p* = 0.004

*Note. ADCS, Alzheimer’s Disease Cooperative Study, possible range 0–78; BIMS, Brief Interview of Mental Status, possible range 0–15; Medi-Cog-R, possible range 0–10; Mini-Cog, possible range 0–5; MoCA, Montreal Cognitive Assessment, possible range 0–30; MTS-R, Medication Transfer Screen-Revised, possible range 0–5; PCST, Performance Assessment of Self-care Skills Checkbook Balancing and Shopping Task*.

## Discussion

There is considerable room for improvement when it comes to evaluating patients for functional cognitive impairments in acute and PAC settings. In these settings, patients are not typically required by hospital staff to complete complex IADL tasks that would potentially identify deficits in functional cognition (Wolf et al., 2019). There is no “gold-standard” to measure everyday abilities and tests that focus on specific cognitive functions such as the MoCA or the BIMS which are currently under consideration by CMS may not be sensitive enough to identify IADL impairments after discharge (Schmitter-Edgecombe et al., 2011). Functional cognitive screening tools could indicate if further evaluation and intervention are needed prior to discharge, however, little evidence exists regarding performance-based functional cognitive screening tools that are brief enough to be practical in acute or post-acute settings. The PCST tasks assess cognitively challenging IADL and reliably differentiate individuals with unimpaired cognitive function from those with MCI (Rodakowski et al., 2014), however, the PCST is a diagnostic assessment and not appropriate for use as a screening test, given that the tasks take 10–15 min to complete and require numerous materials. In addition, the cue-based scoring system requires significant training to assure that the test is administered and scored properly. Quick and easy to administer screening tests with good sensitivity and specificity would benefit clinicians who have limited time to administer assessments like the PCST especially for individuals who do not show obvious signs of impairment.

When evaluating the discriminant validity of the screening tools the Mini-Cog and the Medi-Cog-R unimpaired and impaired groups demonstrated statistically significant differences in scores on the BIMS and the MoCA cognitive screening measures. Individuals impaired on these measures had significantly lower scores on the two brief cognitive assessments supported by CMS. This same pattern was not observed for the MTS-R. Individuals impaired on the MTS-R have significantly lower scores on the MoCA, but essentially equivalent scores on the BIMS, suggesting that the MTS-R requires executive function skills that are assessed by MoCA but not the BIMS. These findings provide support for their ability to rapidly screen for cognitive impairment.

The ADCS is a self-report tool used in Alzheimer’s clinical trials to assess ADL and IADL impairment (Galasko et al., 1997). This assessment is sensitive to the effects of mild and more significant cognitive impairment on functional independence in community-residing older adults. The MTS-R did not significantly discriminate between individuals with and without self-reported ADL/IADL limitations. However, individuals impaired on the Mini-Cog, and the Medi-Cog-R reported significantly lower functional abilities as assessed by the ADCS than those in the unimpaired groups providing additional preliminary support for both the Mini-Cog and Medi-Cog-R regarding ADL/IADL impairment. Individuals impaired on the three screens demonstrated lower scores on other cognitive assessments and reported lower participation in ADL/IADL tasks than the individuals who were unimpaired, providing evidence for discriminant validity.

We confirmed our hypothesis that the combined measure, the Medi-Cog-R, would be superior in classifying an individual’s functional cognitive status and potential for IADL impairment than either the Mini-Cog and the MTS-R alone. On the MTS-R a cutoff score of 4 produced greater specificity than sensitivity, and sensitivity was increased when using a cutoff score of 5 (meaning that any error is indicative of potential impairment). The Mini-Cog by itself exhibited a relatively poor sensitivity in predicting impairment on the PCST, but greater specificity. The Medi-Cog-R, using a cutoff score of 9 (scores of 8 or less are indicative of impairment) produced an optimal combination of sensitivity and specificity. The Medi-Cog-R combines a rapid cognitive measure with a short performance-based measure, the combination of which increased sensitivity to predict impaired performance on the PSCT, a cognitive IADL measure known to be able to discriminate individuals with unimpaired cognitive function from those with MCI. The Medi-Cog has been shown to be a valuable test for medication adherence (Anderson et al., 2008) and our results suggest that the Medi-Cog-R is an appropriate screen in identifying potential risk of deficits in aspects of functional cognition.

We compared the ability of a brief cognitive screening tool, a performance-based screening tool, and their combination to identify individuals with functional cognitive deficits. Our findings suggest that the combined Medi-Cog-R may be superior to both the Mini-Cog and the MTS-R when each is used alone in screening for functional cognitive impairments. The combined Medi-Cog-R most accurately classified individuals as unimpaired or impaired relative to the PCST. Despite its predictive validity for post-hospital support needs, the testing time and training required for accurate administration and scoring of the PCST make it impractical for use as a screening tool in acute or PAC settings. Because the Medi-Cog-R demonstrated adequate ability to classify impairment status as measured by the PCST, there is the potential that clinicians could use the Medi-Cog-R as an initial screen. The Medi-Cog-R has the additional benefit of limited equipment needs and ease of administration and scoring.

Performance-based measures of functional cognition provide health care professionals with an opportunity to directly observe an individual’s ability to complete complex pseudo-IADL tasks, in this case, a medication management task. The revision of the Medi-Cog-R to include the performance-based MTS-R may benefit clinicians by allowing them to not only screen for functional cognitive deficits but also to informally note the individual’s behavior and strategy use while sorting “medications.” With these screening measures, sensitivity is more important than specificity. Screening measures with poor sensitivity may be more accurate in identifying individuals with severe functional cognitive deficits but are less likely to identify individuals with milder forms of impairment that may, none the less, compromise their ability to complete IADLs (Giles et al., 2017). While we did not observe higher sensitivity than specificity in this community sample, the combined measure came closer to this goal and yielded what we think may be an acceptable sensitivity with limited loss of specificity. In a medical setting, impairment on a screening tool such as the Medi-Cog-R would ideally trigger a referral for an in-depth evaluation that would better identify functional cognitive impairments and the potential for such impairments to interfere with IADL that are essential for independent living. Impairment on any of the screening measures examined in this article or the PCST may not warrant the conclusion that functional cognition is impaired broadly, or that the individual will demonstrate deficits in all IADL. Rather, the successful execution of these performance-based measures requires the ability to manage a degree of cognitive complexity that has implications for an individual’s capacity to complete real-world IADL tasks. It is important that if clinicians use these screening tools to briefly assess functional cognition and IADL competency that they also consider these findings in the context of the patient and his/her specific roles and responsibilities. For example, if an individual in acute care or PAC setting performs poorly on the Medi-Cog-R screen, lives alone and has complex dietary restrictions associated with a new diagnosis, the clinician should further determine whether the individual can manage these demands when making or ordering meals. If further observation or evaluation indicates that these abilities are impaired then it is important that proper support is in place for this IADL after discharge.

This study has several limitations. Although we found that the Medi-Cog-R is an adequate functional cognitive screening measure, it requires materials for its administration (4 × 7) section medication organizer, pill bottles, fake pills), which may limit clinical utility for certain populations and settings. In addition, the results should be interpreted with caution as the relative number of individuals with cognitive impairment in our community sample is lower than would be expected in an acute hospital or PAC populations. Further research is warranted to validate these findings and assure generalizability.

### Implications

The Medi-Cog-R may be a useful screening tool to identify individuals at risk of functional cognitive impairment. The use of such a measure could lead to better identification and intervention for individuals at risk whose impairment may have otherwise gone unrecognized. More accurate identification of individuals with functional cognitive impairments may ultimately lead to decreased hospital readmissions and reduced health care costs (RAND Corporation, 2017; Medicare Program, 2019).

## Data Availability Statement

The dataset for this article is not publicly available because the study is still in progress. Requests to access the datasets should be directed to Dorothy Edwards, dfedwards@wisc.edu.

## Ethics Statement

The studies involving human participants were reviewed and approved by Education and Social/Behavioral Science IRB, University of Wisconsin–Madison. The patients/participants provided their written informed consent to participate in this study.

## Author Contributions

TM, GG, DE, and MA-H contributed to the conception and design of the study. TM and MA-H supervised the data collection and scoring. TM organized the database. TM and GG performed the statistical analyses and wrote the first draft of the manuscript. All authors contributed to manuscript revisions, read and approved the submitted version.

## Conflict of Interest

The authors declare that the research was conducted in the absence of any commercial or financial relationships that could be construed as a potential conflict of interest.
